# Body mass index and associated factors among refugees living in North Rhine-Westphalia, Germany: a cross-sectional study

**DOI:** 10.1186/s40795-021-00453-z

**Published:** 2021-08-26

**Authors:** Matthias Hans Belau, Muriel Bassil, Annika Laukamp, Alexander Kraemer

**Affiliations:** 1 Bielefeld University, School of Public Health, Bielefeld, Germany; 2 University Medical Centre Hamburg-Eppendorf, Institute of Medical Biometry and Epidemiology, Hamburg, Germany; 3 University of Michigan, School of Public Health, Ann Arbor, MI USA

**Keywords:** Body mass index, Nutritional status, Health status, Refugees, Germany

## Abstract

**Background:**

This study aims to determine: (i) information on overweight and obesity, represented by body mass index using measured anthropometric data, among refugees living in North Rhine-Westphalia, Germany, (ii) how body mass index changed throughout the migratory journey to Germany, and (iii) factors influencing body mass index.

**Methods:**

The study utilizes data from the FlueGe health study, a cross-sectional study conducted by Bielefeld University. The data was collected between February and November 2018 in which participants were recruited in several cities in North Rhine-Westphalia (*N* = 326). We analyzed differences in body mass index before the escape, upon arrival, and since arrival as well as correlations between body mass index since arrival and explanatory variables using linear regression models.

**Results:**

The overall prevalence of overweight and obesity before the escape (t0), upon arrival (t1) and since arrival (t2) were 55.2% (150/272), 45.6% (133/292) and 54.8% (171/312), respectively, with 16.2% (44/272), 12.0% (35/292) and 16.0% (50/312) being obese. There was a significant change between t0 and t1 (*p* < 0.001), and between t1 and t2 (p < 0.001), but no change over time (between t0 and t2, *p* = 0.713). Results from multivariate linear regression showed that high education, male sex, higher body mass index before the escape, Iranian or Iraqi nationality, and sobriety were the significant factors for body mass index since arrival. However, when focusing on those who have reported weight gain only, higher body mass index before the escape, male sex, and Iraqi nationality were the significant factors.

**Conclusions:**

Overweight and obesity were common among refugees after settlement in Germany. In particular, sociodemographic factors were associated with a higher body mass index since arrival. Thus, it is important to develop and apply nutrition-related intervention programs for adult refugees that are culturally appropriate and tailored to education level and sex.

## Background

In 2015 and 2016, more than a million people fled to Germany from war-affected countries, mainly from Syria, Iraq, Afghanistan, and Central Africa [1]. The health of a migrant and refugee is shaped by their experiences and situations in the migration process [2]. Upon arrival, immigrants are usually healthier than comparable non-migrant populations in the host country [3]. However, the status of a “healthy migrant” changes with time since migration itself can determine health status [4]. Migrants and refugees can be affected by poor living conditions in their new host countries, psychological stresses, and the Westernized lifestyle that is characterized by energy-rich diets and sedentary behavior [4]. This lifestyle affects food consumption [5], and thus the weight and body mass index (BMI) with implications for both physical [6] and mental health [7].

Malnutrition, lack of exercise, and genetic disposition determine the development of overweight (pre-obesity) and obesity [8]. According to the World Health Organization (WHO) classification, overweight is defined by a BMI of 25 kg/m^2^ and higher [9]. A BMI of 30 kg/m^2^ and higher is categorized as obesity [9], which is a risk factor for non-communicable chronic diseases such as hypertension, diabetes mellitus, dyslipidemia, liver disease, kidney disease, sleep apnea, and cardiovascular diseases [6]. Generally, the life expectancy of obese people is lower than that of people of normal weight [10, 11].

Little is known concerning the nutritional status of refugees living in Germany and whether their weight and BMI changed throughout the migratory journey. To the best of our knowledge, only a few studies have examined malnutrition among refugees living in European countries [12–14]. Studies examining the development and determinants of refugees’ nutritional status during settlement in Germany are lacking. A study focusing on African refugees showed that BMI increased significantly from normal to overweight within the year after resettlement in the United States [15].

Several studies show factors influencing weight and BMI, but only a few of them focus on refugees. One study revealed that inadequate fruit and vegetable consumption, physical inactivity, and tobacco consumption are prevalent among Afghan refugees in southern Iran [16], all known risk factors for obesity. Regular physical activity reduces the risk of overweight and obesity in adulthood and the consequent morbidity and mortality associated with it [17]. In addition to exercise, the consumption of fruits and vegetables has been shown to be inversely associated with BMI and directly associated with positive general health outcomes [18]. Studies have also proved that paradoxically, an increase in meal frequency is related to a lower BMI [19]. Nevertheless, prior food scarcity among refugees is positively associated with obesity [20]. Some studies have shown that smoking can also decrease BMI, while smoking cessation stimulates weight gain [21]. On the other hand, alcohol has been found to promote a positive energy balance and contribute to weight gain, increasing BMI [22]. BMI can also be influenced by refugees’ cultural beliefs [23], sociodemographic [24], and socioeconomic status [20]. Considering all this, more studies on the determinants of BMI among adult refugees after settlement are necessary.

The primary goal of our study is to assess valid information regarding overweight and obesity, represented by BMI using measured anthropometric data, among refugees living in North Rhine-Westphalia, Germany, and how BMI changed throughout the migratory journey to Germany. We hypothesized that refugees will lose weight during travel and transit because of their inability to access food and the other struggles they go through during their travel [25]; however, since most refugees in Germany come from the Mediterranean region [26], which is home to one of the healthiest diets [27], their weight will increase after the adaptation of the Westernized lifestyle during settlement, resulting in a higher BMI with time. Our study secondarily examines factors influencing BMI since arrival in Germany, and the results are based on data from the “FlueGe Health Study (FHS)”.

## Methods

### Study settings and participants

The FHS is a cross-sectional study administered by the Forschungskolleg “FlueGe” at Bielefeld University [28]. The FHS aimed to provide health data of refugees from the main countries of origin that contributed to the European refugee crisis in 2015 and 2016 in the region of East Westphalia-Lippe in North Rhine-Westphalia, Germany. The data were collected from February to November 2018. Personal interviews and physical examinations were carried out by trained interviewers. The questionnaire was translated into the following five languages by Kantar Public, a consulting and market research institute: Arabic, Farsi, Kurmanji, English, and German. Participants were recruited from shared and private accommodations in eight different locations in East Westphalia-Lippe. Municipal cooperation partners and social workers helped in the access to potential participants. The FHS included all individuals who were willing to participate (convenience sampling) and signed informed consent. Participants were not eligible for the study if they were younger than 18 years of age, could not speak Arabic, Kurmanji, Farsi, English, or German, or if they have been in Germany for more than 5 years. The concept and design of the FHS are described elsewhere [28].

### Data collection and variables

#### Socio-demographic, lifestyle factors, and health information

After acquiring their consent, eligible participants were interviewed face-to-face by trained interviewers, in the participant’s native language if possible. Socio-demographic information included age, sex (male, female), nationality (Syrians, Afghans, Iraqis, Iranians, African countries, other), length of stay since arrival (< 12, 12–24, 25–36, > 36 months), marital status (unmarried, married, divorced, widowed), employment status (employed, unemployed) and education, which was inquired according to CASMIN [29]. At first, nine educational groups were identified, which resulted in a combination of school and vocational qualifications. For further analysis, the CASMIN index was used to categorize three groups: low (general elementary education and/or basic vocational qualification), medium (intermediate general qualification and/or intermediate vocational qualification), and high education (lower or higher tertiary education). Information on lifestyle factors included exercise (no exercise, less than 120 min, at least 120 min of exercise per week), smoking status (smoker, non-smoker), fruit and vegetable consumption (at least 5 servings, less than 5 servings on an average day), eating habits (more than 4 meals, 4 meals or less per day) as well as alcohol use categorized into the following four groups in accordance with the Epidemiological Survey of Substance Abuse [30]: dangerous consumption (the consumption of five or more alcoholic beverages per day (more than 60 grams (g) of pure alcohol for men and more than 40 g for women), risky consumption (the consumption of three or four alcoholic beverages per day (24-60 g of pure alcohol for men and 12-40 g for women) and low-risk consumption (the consumption of one or two alcoholic beverages per day (< 24 g of pure alcohol for men and < 12 g for women) on at least one day per month. Sobriety (abstinence) was defined as no consumption of alcoholic beverages. Furthermore, participants were asked to state whether a physician diagnosed them with some specific diseases. The following diseases were included in the analysis: depression, diabetes mellitus type II, dyslipidemia, and hypertension.

Following the collection of anthropometric measurements (see below), participants were asked whether their weight had changed (i) during their escape/journey to Germany and (ii) since their arrival in Germany, and if so, how many kilograms (kg) of weight they have gained or lost.

#### Anthropometric measurements

Data was collected on body height, weight, hip and waist circumference, with bare-foot participants in minimal clothing. Height was measured to the nearest centimeter (cm) using portable stadiometers. Weight was assessed to the nearest 0.1 kg using digital scales. Waist and hip circumferences were measured in cm to the nearest 0.1 cm, using inextensible anthropometric tape. Waist-to-hip ratio, abdominal obesity, and BMI were determined using definitions by the WHO [9, 31].

#### Body mass index

Body mass index was calculated by dividing a person’s weight in kg by his or her height, in meters squared (m^2^) [9]. The number generated from this equation was considered the individual’s BMI since arrival. Furthermore, BMI upon arrival was calculated by measured weight ± reported weight gained or lost since arrival (in kg) / measured height^2^ (in m) and BMI before the escape was measured by measured weight ± reported weight gained or lost since arrival ± weight gained or lost during escape (in kg) / measured height^2^ (in m). Finally, BMI was categorized into underweight (BMI < 18.5 kg/m^2^), normal weight (BMI 18.5–24.9 kg/m^2^), pre-obesity (overweight, BMI 25.0–29.9 kg/m^2^), or obesity (class I/II/III, BMI ≥30.0 kg/m^2^) in accordance with the WHO classification for adults [9].

#### Waist-to-hip ratio, abdominal obesity

The waist-to-hip ratio was calculated as waist circumference in cm divided by hip circumference in cm [31]. Abdominal obesity was defined as a waist-to-hip ratio above 0.90 for males and 0.85 for females [31].

### Statistical analyses

Statistical analyses were performed using STATA version 15.1. In the first analysis step, the data were evaluated descriptively. Differences in BMI before the escape, upon arrival, and since arrival by sex and age group (18–24, 25–29, 30–39, and 40+ years) were analyzed using the Chi-squared test. Differences in BMI over time were analyzed using Wilcoxon signed-rank test. Linear regression using the backward elimination technique [32] with bias-corrected and accelerated (BCa) confidence interval (bootstrapping, based on 5000 samples) [33] was applied to assess the potential predictors of BMI since arrival. Cases presenting a missing value for at least one of the modeling variables were excluded from the analyses (listwise exclusion). In the regression model, the dependent variable was BMI since arrival and the independent variables were age, sex, nationality (as dummy variables (DV) to indicate the absence or presence of categorical effect [32]), marital status (DV), education (DV), employment status, length of stay since arrival (DV), exercise (DV), smoking status, alcohol use (DV), fruit and vegetable consumption, eating habits, health conditions (DV), waist-to-hip ratio, abdominal obesity, and BMI before the escape. All the independent variables were entered into the equation first (full model) and removed one at a time, starting with the variable with the highest *p*-value. We set a *p*-value threshold at 0.05 to determine when to stop removing variables from the model. Effects with *p*-values < 0.05 were considered statistically significant. Attention was paid to linear regression assumptions, interactions among variables of interest, and plausibility of the identified effects. The model finding procedure was repeated in a sub-sample with cases that reported weight gain since arrival.

## Results

Table 1 summarizes the characteristics of the study population (*n* = 326). The majority were male, most immigrated from Middle Eastern countries, and the median age was 30.0 years. The mean (SD) BMI before escape (t0), upon arrival (t1), and since arrival (t2) were 25.8 (4.6), 24.8 (4.7), and 25.9 (4.8) kg/m^2^ respectively. There was a difference in mean (SD) BMI between sexes before the escape to Germany (men 25.4 (4.0) kg/m^2^, women 26.9 (5.8) kg/m^2^, *p* = 0.024), upon arrival to Germany (men 24.5 (4.2) kg/m^2^, women 25.8 (5.8) kg/m^2^, *p* = 0.034) and since the arrival to Germany (men 25.2 (4.0) kg/m^2^, women 28.1 (6.2) kg/m^2^, *p* < 0.001), with females having a higher BMI in all three stages. Nevertheless, there was a significant change in BMI between t0 and t1 (p < 0.001), and between t1 and t2 (p < 0.001), but no changes in BMI over time (between t0 and t2, *p* = 0.713).

**Table 1:** Characteristics of the study population (*N* = 326)

		Overall		Male		Female	
		n	%	n	%	n	%
**Age**	Mean (SD)	32.4 (11.0)		31.9 (11.2)		33.8 (10.4)	
**Sex**		326	100.0	238	73.0	88	27.0
**Nationality**	Syrians	132	40.5	96	40.3	36	40.9
	Afghans	42	12.9	33	13.9	9	10.2
	Iraqis	79	24.2	56	23.5	23	26.1
	Iranians	19	5.8	14	5.9	5	5.7
	African countries^a^	23	7.1	14	5.9	9	10.2
	Other^b^	30	9.2	24	10.1	6	6.8
	*Missing values*	*1*	*0.3*	*1*	*0.4*	*0*	*0.0*
**Marital status**	Unmarried	145	44.5	122	51.3	23	26.1
	Married	154	47.2	103	43.3	51	58.0
	Divorced	18	5.5	9	3.8	9	10.2
	Widowed	5	1.5	2	0.8	3	3.4
	*Missing values*	*4*	*1.2*	*2*	*0.8*	*2*	*2.3*
**Education**	Low	135	41.4	93	39.1	42	47.7
	Medium	127	39.0	97	40.8	30	34.1
	High	63	19.3	48	20.2	15	17.1
	*Missing values*	*1*	*0.3*	0	0.0	1	1.1
**Employment status**	Employed	33	10.1	33	13.9	0	0.0
	Unemployed	284	87.1	199	83.6	85	96.6
	*Missing values*	*9*	*2.8*	6	2.5	3	3.4
**Length of stay since arrival**	*< 12 months*	42	12.9	21	8.8	21	23.9
	*12–24 months*	32	12.9	22	9.2	10	11.4
	*25–36 months*	168	51.5	139	58.4	29	33.0
	*> 36 months*	80	24.5	54	22.7	26	29.6
	*Missing values*	*4*	*1.2*	2	0.8	2	2.3
**Exercise (sport)**	No exercise	176	54.0	114	47.9	62	70.5
	< 120 min per week	102	31.8	83	35.2	19	22.4
	≥120 min per week	43	13.4	39	16.5	4	4.7
	*Missing values*	*5*	*1.5*	2	0.8	3	3.4
**Smoking status**	Smoker	180	55.2	151	63.5	29	33.0
	Non-smoker	141	43.3	85	35.7	56	63.6
	*Missing values*	*5*	*1.5*	2	0.8	3	3.4
**Alcohol use**	Sobriety/abstinence	187	57.4	119	50.0	68	77.3
	Low-risk consumption^c^	81	24.9	72	30.3	9	10.2
	Risky consumption^d^	35	10.7	28	11.8	7	8.0
	Dangerous consumption^e^	11	3.4	10	4.2	1	1.1
	*Missing values*	*12*	*3.7*	9	3.8	3	3.4
**Fruit & vegetable consumption**	≥5 servings per day	28	8.6	18	7.6	10	11.4
	< 5 servings per day	290	89.0	214	90.0	76	86.4
	*Missing values*	*8*	*2.5*	6	2.5	2	2.3
**Eating habits**	> 4 meals per day	70	21.5	54	22.7	16	11.4
	≤4 meals per day	253	77.6	183	76.9	70	86.4
	*Missing values*	*3*	*0.9*	1	0.4	2	2.3
**BMI before escape**	Mean (SD)	25.8 (4.6)		25.4 (4.0)		26.9 (5.8)	
	*Missing values*	*53*	*16.3*	34	14.3	19	21.6
**BMI upon arrival**	Mean (SD)	24.8 (4.7)		24.5 (4.2)		25.8 (5.8)	
	*Missing values*	*30*	*9.2*	19	8.0	11	12.5
**BMI since arrival**	Mean (SD)	25.9 (4.8)		25.2 (4.0)		28.1 (6.2)	
	*Missing values*	*14*	*4.3*	8	3.4	6	6.8
**Waist-to-hip ratio since arrival**	Mean (SD)	0.90 (0.1)		0.91 (0.1)		0.87 (0.1)	
	*Missing values*	*15*	*4.6*	9	3.8	6	6.8
**Abdominal obesity**	Yes	182	55.8	130	54.6	52	59.1
	*Missing values*	*15*	*4.6*	9	3.8	6	6.8
**Health conditions (self-report)**	Depression	47	14.4	34	14.3	13	14.8
	*Missing values*	*5*	*1.5*	5	2.1	0	0.0
	Diabetes mellitus (type II)	12	3.7	6	2.5	6	6.8
	*Missing values*	*4*	*1.2*	4	1.7	0	0.0
	Dyslipidemia	16	4.9	11	4.6	5	5.7
	*Missing values*	*7*	*2.2*	5	2.1	2	2.3
	Hypertension	21	6.4	16	6.7	5	5.7
	*Missing values*	*5*	*1.5*	5	2.1	0	0.0

n: quantity; %: proportion; SD: standard deviation

^**a**^ Algeria (8.7%), Eritrea (13.0%), Nigeria (26.1%), Somalia (17.4%), Ghana (13.0%), Guinea (4.4%), Morocco (8.7%), Egypt (8.7%); ^**b**^ Azerbaijan (3.3%), Bangladesh (20.0%), Georgia (3.3%), India (3.3%), Lebanon (10.0%), Pakistan (16.7%), Palestine (6.7%), Russia (6.7%), Tajikistan (3.3%), Turkey (10.0%), stateless (16.7%); ^**c**^ consumption of one or two alcoholic beverages (< 24 g for men and < 12 g pure alcohol for women per day) on at least one day in a month; ^**d**^ consumption of three or four alcoholic beverages (24-60 g for men and 12-40 g pure alcohol for women per day) on at least one day in a month; ^**e**^ consumption of five or more alcoholic beverages (more than 60 g for men and 40 g pure alcohol for women per day) on at least one day in a month

Table 2 provides detailed information on sociodemographic and lifestyle factors stratified by BMI categories (since the arrival to Germany). The overall prevalence of overweight and obesity was 54.8% (men 49.6%, women 69.5%) with 16.0% (men 10.4%, women 31.7%) categorized as having obesity. Mean (SD) age increased with higher BMI category from 21.3 (3.1) kg/m^2^ (underweight) to 37.6 (11.9) kg/m^2^ (obesity). In addition to that, most participants with high education were categorized as overweight and obese, and the majority of employed participants were of normal weight.

**Table 2:** Sociodemographic and lifestyle factors of study population stratified by BMI categories since arrival to Germany (N = 326)

		Underweight		Normal weight		Pre-obesity (overweight)		Obesity class I/II/III	
		n	%	n	%	n	%	n	%
**Age**	Mean (SD)	21.3 (3.1)		29.7 (9.8)		34.7 (10.9)		37.6 (11.9)	
**Sex**	Male	7	3.0	109	47.4	90	39.1	24	10.4
	Female	3	3.7	22	26.8	31	37.8	26	31.7
**Nationality**	Syrians	5	3.9	50	38.8	53	41.1	21	16.3
	Afghans	1	2.4	21	50.0	15	35.7	5	11.9
	Iraqis	2	2.7	27	37.0	29	39.7	15	20.6
	Iranians	0	0.0	8	42.1	11	57.9	0	0.0
	African countries^a^	1	4.6	10	45.5	5	22.7	6	27.3
	Other^b^	1	3.9	14	53.9	8	30.8	3	11.5
**Marital status**	Unmarried	8	5.8	74	53.2	50	36.0	7	5.0
	Married	2	1.3	52	34.7	59	39.3	37	24.7
	Divorced	0	0.0	3	16.7	10	55.6	5	27.8
	Widowed	0	0.0	2	40.0	2	40.0	1	20.0
**Education**	Low	4	3.2	49	38.9	48	38.1	25	19.8
	Medium	6	4.9	59	48.0	42	34.2	16	13.0
	High	0	0.0	23	36.5	31	49.2	9	14.3
**Employment status**	Employed	0	0.0	23	71.9	8	25.0	1	3.1
	Unemployed	10	3.7	104	38.4	109	40.2	48	17.7
**Length of stay since arrival**	*< 12 months*	0	0.0	16	41.0	19	48.7	4	10.3
	*12–24 months*	2	6.9	116	55.2	6	20.7	5	17.2
	*25–36 months*	6	3.7	72	43.9	61	37.2	25	15.2
	*> 36 months*	2	2.6	26	33.8	35	45.5	14	18.2
**Exercise (sport)**	No exercise	6	3.5	62	36.3	70	40.9	33	19.3
	< 120 min per week	2	2.1	46	47.4	34	35.1	15	15.5
	≥120 min per week	1	2.4	23	54.8	16	38.1	2	4.8
**Smoking status**	Smoker	5	3.0	44	32.8	56	41.8	30	22.4
	Non-smoker	4	2.8	86	48.9	65	36.9	20	11.4
**Alcohol use**	Sobriety/abstinence	7	3.9	73	40.8	68	38.0	31	17.3
	Low-risk consumption^c^	1	1.3	34	43.0	31	39.2	13	16.5
	Risky consumption^d^	2	5.7	17	48.6	13	37.1	3	8.6
	Dangerous consumption^e^	0	0.0	3	27.3	5	45.5	3	27.3
**Fruit & vegetable consumption**	≥5 servings per day	1	3.6	7	25.0	14	50.0	6	21.4
	< 5 servings per day	9	3.2	120	43.0	106	38.0	44	15.8
**Eating habits**	> 4 meals per day	2	3.0	31	47.0	25	37.9	8	12.1
	≤4 meals per day	8	3.3	100	40.7	96	39.0	42	17.1

n: quantity; %: proportion; SD: standard deviation

^**a**^ Algeria, Eritrea, Nigeria, Somalia, Ghana, Guinea, Morocco, Egypt; ^**b**^ Azerbaijan, Bangladesh, Georgia, India, Lebanon, Pakistan, Palestine, Russia, Tajikistan, Turkey, stateless; ^**c**^ consumption of one or two alcoholic beverages (< 24 g for men and < 12 g pure alcohol for women per day) on at least one day in a month; ^**d**^ consumption of three or four alcoholic beverages (24-60 g for men and 12-40 g pure alcohol for women per day) on at least one day in a month; ^**e**^ consumption of five or more alcoholic beverages (more than 60 g for men and 40 g pure alcohol for women per day) on at least one day in a month

Table 3 shows bivariate associations between BMI categories (before the escape, upon and since the arrival to Germany) and age group, overall and stratified by sex. At all times, there was a significant difference among age groups. Before the escape to Germany, most participants in younger age groups reported a normal weight, while most participants in older age groups were categorized as having overweight. Throughout the journey, many participants experienced weight changes. Overall, across age groups, most participants in younger age groups reported a normal weight upon arrival to Germany. In older age groups, a decrease in the proportion of overweight and obesity was found. At the time of data collection, most participants aged 29 and under had a normal weight, except for females aged 25–29. As for most participants aged 30 years and older, BMI since the arrival to Germany was categorized as overweight.

**Table 3:** BMI categories before the escape, upon and since arrival to Germany, stratified by sex and age group (N = 326)

	Age group								***p***-value
	18–24		25–29		30–39		40+		
	n	%	n	%	n	%	n	%	
**BMI before escape**									
** Overall**									< 0.001
Underweight	8	11.4	2	3.3	0	0.0	0	0.0	
Normal weight	40	57.1	28	45.9	24	30.8	20	31.8	
Pre-obesity (overweight)	18	25.7	22	36.1	36	46.2	30	47.6	
Obesity class I/II/III	4	5.7	9	14.8	18	23.1	13	20.6	
** Men**									0.001
Underweight	2	3.7	2	4.0	0	0.0	0	0.0	
Normal weight	36	66.7	27	54.0	15	27.8	15	33.3	
Pre-obesity (overweight)	14	25.1	15	30.0	28	51.9	22	48.9	
Obesity class I/II/III	2	3.7	6	12.0	11	20.4	8	17.8	
** Women**									0.002
Underweight	6	37.5	0	0.0	0	0.0	0	0.0	
Normal weight	4	25.0	1	9.1	9	37.5	5	27.8	
Pre-obesity (overweight)	4	25.0	7	63.6	8	33.3	8	44.4	
Obesity class I/II/III	2	12.5	3	27.3	7	29.2	5	27.8	
**BMI upon arrival**									
**Overall**									< 0.001
Underweight	10	13.7	2	3.0	0	0.0	0	0.0	
Normal weight	48	65.8	32	48.5	37	43.5	30	44.1	
Pre-obesity (overweight)	11	15.1	25	37.9	36	42.4	26	38.2	
Obesity class I/II/III	4	5.5	7	10.6	12	14.1	12	17.7	
**Men**									< 0.001
Underweight	4	7.0	2	3.8	0	0.0	0	0.0	
Normal weight	43	75.4	30	56.6	25	43.1	18	38.3	
Pre-obesity (overweight)	7	12.3	16	30.2	27	46.6	20	42.6	
Obesity class I/II/III	3	5.3	5	9.4	6	10.3	9	19.2	
**Women**									< 0.001
Underweight	6	37.5	0	0.0	0	0.0	0	0.0	
Normal weight	5	31.3	2	15.4	12	44.4	12	57.1	
Pre-obesity (overweight)	4	25.0	9	69.2	9	33.3	6	28.6	
Obesity class I/II/III	1	6.3	2	15.4	6	22.2	3	14.3	
**BMI since arrival**									
**Overall**									< 0.001
Underweight	9	11.3	1	1.4	0	0.0	0	0.0	
Normal weight	50	62.5	30	42.9	26	29.6	25	33.8	
Pre-obesity (overweight)	17	21.3	28	40.0	44	50.0	32	43.2	
Obesity class I/II/III	4	5.0	11	15.7	18	20.5	17	23.0	
**Men**									< 0.001
Underweight	6	9.4	1	1.8	0	0.0	0	0.0	
Normal weight	43	67.2	29	51.8	20	32.8	17	34.7	
Pre-obesity (overweight)	14	21.1	20	35.7	33	54.1	23	46.9	
Obesity class I/II/III	1	1.6	6	10.7	8	13.1	9	18.4	
**Women**									0.014
Underweight	3	18.8	0	0.0	0	0.0	0	0.0	
Normal weight	7	43.8	1	7.1	6	22.2	8	32.0	
Pre-obesity (overweight)	3	18.8	8	57.1	11	40.7	9	36.0	
Obesity class I/II/III	3	18.8	5	35.7	10	37.0	8	32.0	

n: quantity; %: proportion

Table 4 displays the final model between BMI since arrival and independent variables, including all participants possible (*n* = 265), and Table 5 only included those who reported weight gain since arrival (*n* = 176). After eliminating the independent variables that were not considered significant, both regression models included being male, being Iraqi, and BMI before escape as independent variables. The model with cases that reported weight gain additionally included abstinence from alcohol, high education, and being Irani as independent variables.

**Table 4:** Linear regression model between BMI since arrival and independent variables (backward elimination), bias-corrected and accelerated bootstrapped confidence interval based on 5000 samples (N = 265)

BMI since arrival, overall	β	95% CI		***p***-value
		Lower	Upper	
Education, high (vs. medium, low)	1.28	0.24	2.33	0.016
Male sex	−1.72	− 2.59	− 0.85	< 0.001
BMI before escape	0.80	0.71	0.89	< 0.001
Iranian	− 1.79	−3.07	− 0.50	0.006
Iraqi	−0.97	−1.68	− 0.25	0.008
Alcohol abstinence^a^ (vs. alcohol use)	− 0.81	− 1.56	− 0.06	0.034
Intercept	6.97	4.52	9.41	< 0.001
*Coefficient of determination (R*^*2*^*):*	*0.664*			
*Adjusted R*^*2*^*:*	*0.656*			
*F-value:*	*84.9, p < 0.001*			

β: regression coefficient, CI: confidence interval

^a^ no consumption of alcoholic beverages on at least one day in a month

**Table 5:** Linear regression model between BMI since arrival and independent variables (backward elimination), sub-sample with cases that reported weight gain, bias-corrected and accelerated bootstrapped confidence interval based on 5000 samples (N = 176)

BMI since arrival, weight gained since arrival	β	95% CI		***p***-value
		Lower	Upper	
BMI before escape	0.86	0.74	0.97	< 0.001
Male sex	−0.97	−1.91	−0.03	0.042
Iraqi	−0.94	−1.67	−0.21	0.012
Intercept	5.68	2.88	8.48	< 0.001
*Coefficient of determination (R*^*2*^*):*	*0.676*			
*Adjusted R*^*2*^*:*	*0.671*			
*F-value:*	*119.9, p < 0.001*			

β: regression coefficient, CI: confidence interval

## Discussion

Our results suggest that overweight and obesity are common in refugees before the escape, upon the arrival, and since the arrival to Germany, which is comparable to other studies [34, 35]. Furthermore, the results showed that refugees tend to lose weight during their escape and gain weight since their arrival, but without a significant change over time. We assume that loss in weight during escape is partly due to displacement or migration stressors (followed by substantial changes in diet) and once they are settled, refugees return to their initial weight. Compared to the general German population, a higher proportion of the females in the population studied is considered overweight or obese [36]. In contrast, a higher proportion of males is considered at normal weight compared to the general German population [36].

Moreover, our results demonstrate that men showed a lower BMI since arrival generally (Table 4) and among refugees that have gained weight specifically (Table 5). Females are more vulnerable to gain weight due to the influence of gonadal steroids on body composition and appetite, and behavioral, socio-cultural, and chromosomal factors [37]. The females in the population studied also engaged in less physical activity than males. A study focusing on Iraqi refugees in Michigan showed that after a year of settlement in the US, female refugees gained more weight than males [34]. These findings show the need for tailoring an obesity prevention program separately for each sex.

A study assessing stressors and body weight development of Iraqi refugees in Michigan showed an increase in smoking and alcohol consumption after one year of arrival [38]. This study also showed that there were no differences in BMI changes over the first year between drinkers and non-drinkers at the time of arrival. In our study, sobriety or abstinence from alcohol was associated with a lower BMI. This can simply be related to the fact that alcohol is a highly caloric beverage that can disrupt metabolic function and lead to weight gain [39].

A study assessing predictors of BMI in Jordan showed that Syrian refugees had a significantly higher BMI than Iraqis [40]. This was explained by the fact that Syrians represented 80% of their study population and Iraqis might be underrepresented. Their study also showed that a high percentage of refugees had a low physical activity level, which can affect BMI. As a result, this suggests that a high percentage of Syrian refugees were physically inactive, contributing to a high BMI [40]. This is in accordance with our study since most refugees in our study population did not engage in any exercise and only a few exercised the recommended 120 minutes per week; however, physical activity was not shown to be significantly related to BMI in our data. As far as our knowledge, no literature could explain why Iraqis and Iranians in our study had a significantly lower BMI than other nationalities at the point of data collection. Iraqis represented one-fourth of our study population while Iranians represented almost 6% of the population. Dietary assessment and consultation should be tailored to different nationalities instead of having one universal assessment for all refugees in Germany because refugees from different nationalities have different eating habits and dietary patterns, affecting their weight and BMI.

In previous studies, higher education is shown to be associated with lower BMI since highly educated people are more knowledgeable about health risks associated with overweight and obesity and the benefits of a healthy diet and physical activity [40]. In contrast, our study showed a higher BMI with attained high education. This can be explained by the fact that better education can lead to a better job and thus higher income and food accessibility; however, unlike previous research [40], employment status was not significantly related to BMI. Perhaps also cultural attitudes towards body shape and weight play a role (body perception). Further data collection and analysis are needed.

In addition, having a higher BMI before the escape was significantly related to a higher BMI since arrival. As we mentioned before, acute stress due to (forced) migration may reduce food intake and hence body weight. In our study, we observed weight loss during the escape and regain since arrival without a significant change over time. Thus, the return to initial weight could be described as a form of the “yo-yo effect” [41].

### Limitations

This study faces a few limitations. We utilized data from the FHS, and selection bias might be an issue because individuals who participated in the FHS were self-selected. This means that individuals who were not comfortable with their weight might have decided not to participate in the FHS or decided to opt out of the anthropometric measurements. In addition to that, the research questions for this study were developed after the data was collected so we lack some important information that could be helpful to further understand the relationship between BMI changes and the Westernized lifestyle. Asking about dietary patterns in their home country, during the escape journeys, and since their arrival might be beneficial to further understand the changes in eating behavior and weight among refugees. Another limitation is that our studied population had a short duration of stay in Germany with a maximum of 5.5 years (and a minimum of 1 month). A longitudinal study following the refugees’ BMI and eating behaviors with time could be elucidating for further analysis. To calculate BMI before escape and directly after the escape, weight was assessed based on self-reported weight gained or lost which may not be very accurate. Women tend to underreport their weight while men tend to overreport their weight unless they were obese [42]. A study comparing self-reported and measured BMI among Bhutanese refugee women resettled in the U.S. found that over half of the women were overweight or obese, with only 8% self-reporting being overweight or obese [43]. Both sexes tend to overreport physical activity [44] which may lead to information bias. If the information about weight gained or lost was not reported correctly, then misclassification of individuals in the wrong BMI category can lead to an inaccurate prevalence of overweight and obesity before escape and upon arrival. Participants were asked, following the anthropometric measurements’ collection, whether their weight changed (i) during escape/journey to Germany and (ii) since arrival in Germany. Some participants were unable to provide information on their weight before escape (*n* = 50) and upon arrival (*n* = 30), and information bias cannot be ruled out. Nevertheless, self-reported weight change throughout the migratory process still provides important information, and the literature supports the use of self-reported weight data to calculate BMI for weight classification purposes when anthropometric measurements are not feasible [45]. Studies comparing self-reported and measured anthropometric measurements in an adult population showed good agreement across different sociodemographic groups [46, 47]. Other potential examples of information bias include the reporting of diagnosed disorders such as depression, hypertension, diabetes, and others. Furthermore, since our data only included a convenience sample of 326 refugees from East Westphalia-Lippe in North Rhine-Westphalia results cannot be generalized to all refugees in North Rhine-Westphalia and Germany. Finally, it is important to note that the refugees that were able to come to Germany from Africa and the Middle East might be in relatively better health than refugees who settled in neighboring countries of their home country. To truly understand the effects of a Westernized lifestyle on the weight of refugees coming from non-Western countries, a comparative study with refugees coming from the same country but settling in non-Western countries might be valuable. Despite its limitations, the present study provides relevant data on the nutritional status of adult refugees living in North Rhine-Westphalia, Germany. Key strengths of our study include its multiethnic refugee sample, the inclusion of both men and women, face-to-face interviews in participants’ native languages, and comprehensive information on sociodemographic and lifestyle factors across BMI levels. Another strength is that we did not focus on overweight and obesity alone but assessed correlations between BMI since arrival and explanatory variables, as well.

## Conclusions

This is the first study investigating information related to overweight and obesity by BMI using reported and measured anthropometric data among adult refugees living in North Rhine-Westphalia, Germany. Our study provides information on changes in nutritional status in the migration process. In conclusion, overweight and obesity were common among refugees after settlement in Germany. There was a significant change between BMI before escape and BMI upon arrival, and between BMI upon arrival and BMI since arrival, but no change over the whole period. In linear regression analysis, sex, nationality, education, non-alcohol use, and reported BMI before escape were associated with BMI since arrival. It is therefore important to develop and apply culturally appropriate nutrition-related intervention programs for adult refugees that are tailored to their education level and sex.

## Data Availability

The data that support the findings of this study is available at Bielefeld University, but restrictions apply to the availability of these data, which were used under license for the current study, and so are not publicly available. Data is however available from the authors upon reasonable request and with permission of Bielefeld University.

## Abbreviations

BCa: Bias-corrected and accelerated
BMI: Body mass index
DV: Dummy variable
FHS: FlueGe Health Study
WHO: World Health Organization
